# A nuclease-polymerase chain reaction enables amplification of probes used for capture-based DNA target enrichment

**DOI:** 10.1093/nar/gkz870

**Published:** 2019-10-10

**Authors:** Ka Wai Leong, Fangyan Yu, Viktor A Adalsteinsson, Sarah Reed, Gregory Gydush, Ioannis Ladas, Jiang Li, Kelan G Tantisira, Gerassimos Mike Makrigiorgos

**Affiliations:** 1 Department of Radiation Oncology, Dana-Farber Cancer Institute and Brigham and Women's Hospital, Harvard Medical School, Boston, MA 02115, USA; 2 The Broad Institute of MIT and Harvard, Cambridge, MA 02142, USA; 3 The Channing Division of Network Medicine, Department of Medicine, Brigham and Women's Hospital and Harvard Medicine School, Boston, MA 02142, USA

## Abstract

DNA target enrichment via hybridization capture is a commonly adopted approach which remains expensive due in-part to using biotinylated-probe panels. Here we provide a novel isothermal amplification reaction to amplify rapidly existing probe panels without knowledge of the sequences involved, thereby decreasing a major portion of the overall sample preparation cost. The reaction employs two thermostable enzymes, BST-polymerase and duplex-specific nuclease DSN. DSN initiates random ‘nicks’ on double-stranded-DNA which enable BST to polymerize DNA by displacing the nicked-strand. Displaced strands re-hybridize and the process leads to an exponential chain-reaction generating biotinylated DNA fragments within minutes. When starting from single-stranded-DNA, DNA is first converted to double-stranded-DNA via terminal-deoxynucleotidyl-transferase (TdT) prior to initiation of BST–DSN reaction. Biotinylated probes generated by **T**dT–**B**ST–**D**SN (**TBD**) reactions using panels of 33, 190 or 7186 DNA targets are used for hybrid-capture-based target enrichment from amplified circulating-DNA, followed by targeted re-sequencing. Polymerase-nuclease isothermal-chain-reactions generate random amplified probes with no apparent sequence dependence. One round of target-capture using TBD probes generates a modest on-target sequencing ratio, while two successive rounds of capture generate >80% on-target reads with good sequencing uniformity. TBD-reactions generate enough capture-probes to increase by approximately two to three orders-of-magnitude the target-enrichment experiments possible from an initial set of probes.

## INTRODUCTION

With the rapidly decreasing cost of sequencing, methods for efficient and low-cost sample preparation become increasingly important. Target enrichment prior to targeted re-sequencing comprises a major part of the effort and cost involved in sample preparation (1,2). Target enrichment via hybridization capture is advantageous when large panels of DNA targets need be sequenced and is adopted commonly both for human as well as non-human DNA such as whole viral genomes (3). Additionally, hybridization capture allows the expansion of knowledge of non-reference organisms and ancient genomes and provides a better understanding of metagenomic DNA (4). Such hybrid capture relies on the availability of biotinylated probe panels, which comprise a significant portion in the overall cost of sample preparation for sequencing. Approaches employing biotinylated polymerase chain reaction (PCR) products as capture probes for small numbers of DNA targets have also been described (5).

Here we provide a method to amplify any available panel of probes without requiring information on the sequences involved. An isothermal DNA amplification reaction, BST–DSN is presented using two thermostable enzymes, BST DNA polymerase (BST) and duplex-specific nuclease (DSN) which has minimal activity on single stranded DNA (6,7) while it generates single strand breaks on double stranded DNA with no apparent sequence preference (6).

**Figure 1.**
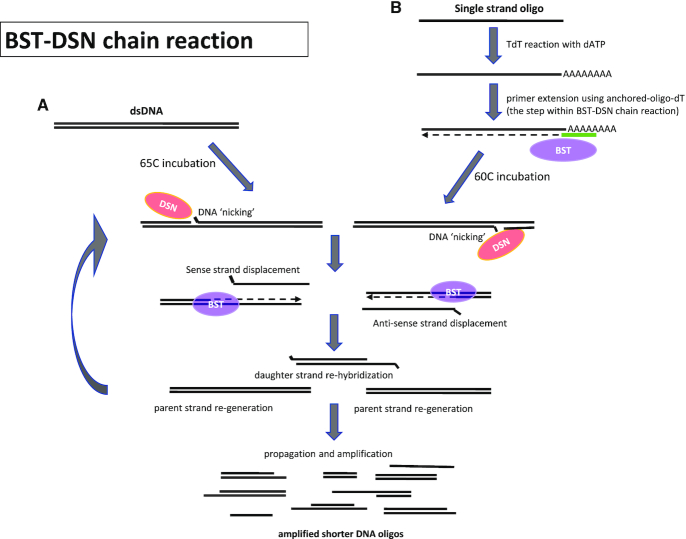
Diagram illustrating the BST–DSN reaction process. (**A**) When dsDNA is used as input in a BST–DSN reaction, the nuclease DSN nicks one strand of dsDNA to create a recognition site for BST polymerase which then synthesizes a complement of the opposite DNA strand while displacing the parent strand. The displaced-sense (or anti-sense) DNA strands subsequently can re-hybridize to complementary strands and form daughter dsDNA. Subsequent DSN nicking and BST amplification generated an exponential amplification of daughter dsDNA while progressively reducing the resulting DNA size. (**B**) When single stranded DNA (ssDNA) or long oligonucleotides are used as input in BST–DSN reaction, the ssDNA is first subjected to a TdT reaction in the presence of dATP to generate a poly-A tail on the 3′ end. The unpurified TdT product is then used as input in a BST–DSN reaction in the presence of an anchored-oligo-dT which is extended by BST to create dsDNA as a first step in the reaction.

When dsDNA is applied as starting material in a BST–DSN reaction, DSN produces random single strand breaks (nicks, Figure 1). The nicks are then recognized by BST which initiates strand displacement DNA synthesis and re-generates the original dsDNA molecule. The displaced DNA may re-hybridize with displaced DNA from an opposite strand of the DNA target and forms a daughter dsDNA which participates in new BST–DSN reactions. The BST–DSN chain reaction produces short DNA fragments from dsDNA template and reaches completion within minutes.

When ssDNA is applied as starting material in BST–DSN reaction, an additional step is included to convert ssDNA to dsDNA (Figure 1). Terminal deoxynucleotidyl transferase (TdT) is employed to synthesize a poly-A tail on the 3′end of ssDNA. Products from TdT reaction are then used in BST–DSN reactions that include an anchor-oligo-dT to generate dsDNA from the poly-A tails, following which the chain amplification by BST–DSN takes place. By including biotinylated nucleotides in the **T**dT–**B**ST–**D**SN (TBD) reaction, the amplification reaction produces copious amounts of biotinylated probes that can be used directly as ‘baits’ for target enrichment from human genomic DNA; thereby greatly increasing the number of reactions that can be performed from an initial input of capture probes and reducing the overall sample preparation cost. We validate the TBD reaction-generated capture probes using either a custom-made panel of PCR products as input DNA, or commercially available sets of long oligonucleotides (‘ultramers’) covering 33, 190 or 7816 genomic targets of interest, and by performing target enrichment and sequencing from amplified cell-free circulating DNA (cfDNA).

## MATERIALS AND METHODS

### Cell-free circulating DNA (cfDNA) and ligation-mediated PCR (LMPCR)

cfDNA from healthy volunteers were obtained from Brigham and Women's Hospital under Institutional Review Board approval. cfDNA was isolated from plasma using the QIAamp Circulating Nucleic Acids Kit (Qiagen) and was quantified on a Qubit 3.0 fluorometer using a dsDNA HS assay kit (Thermo Fisher Scientific). cfDNA was then subjected to end-repair and adaptor ligation (NEBNext Ultra II DNA Library Prep Kit, New England Biolabs, NEB) followed by 15 cycles of amplification via ligation-mediated PCR (LMPCR) using Q5 DNA polymerase (NEB).

LMPCR product similarly obtained by using cfDNA from cancer patient #295 was also used for this study, under Institutional Review Board approval. Somatic mutations in this sample had been previously identified via exome sequencing of the primary tumor, as well as via exome sequencing of cfDNA (7). To generate low mutation allelic frequency from this sample, a 20-fold dilution into LMPCR product obtained from healthy volunteers’ cfDNA was applied.

### Polymerase chain reaction (PCR) amplification of gene targets

PCR reactions targeting p53, NOP14, MTMR4, ZPLD1, CDHR3, GMPR, CACNA1I, OR2S2, AGHGEF12, CACNA1C, SAMDA4, KRAS, BRAF and NGLY1 were performed on CFX Connect^Tm^ real-time PCR machine (Bio-Rad Laboratories) using Phusion High-Fidelity DNA polymerase (Thermo Fisher Scientific) per the protocol provided in Supplementary Table S1. All primers were synthesized by IDT (Integrated DNA Technologies, IDT) using primers depicted in Supplementary Table S2.

### BST–DSN reaction using PCR products as input

BST 2.0 DNA polymerase (BST) and DSN were purchased from NEB and Sapphire North America, respectively. BST–DSN reaction was conducted. PCR products were mixed in a 10 μl final volume of BST–DSN reaction master mix per the protocol provided on Supplementary Table S3. BST–DSN reaction was conducted in a Cepheid Smart cycler II thermocycler set at a constant 65°C as shown on Supplementary Table S3. The reaction was followed in real time by including a DNA intercalating dye, LCGreen (BioFire Diagnostics) in the reaction and reading the fluorescent signal in 12 s ‘cycles’. A QIAquick™ Nucleotide Removal Kit (Qiagen) was used to purify the BST–DSN products and the size of BST–DSN products was analyzed by Agilent DNA Chip 1000 (Agilent).

### BST–DSN reaction using single strand DNA (ssDNA) as input

Custom long oligonucleotide ‘ultramer’ probes (33-plex, Panel A) were obtained from IDT (Integrated DNA Technologies). NEBNext Direct™ Cancer hotspot panel (Panel B) and xGen Pan Cancer™ panel (Panel C) were purchased from NEB and IDT, respectively.

TdT (from NEB) reaction was performed on a thermocycler (Mastercycler Nexus, Eppendorf) to generate poly-adenines at the 3′end of ssDNA prior to BST–DSN reaction. The protocol is described in Supplementary Table S3. The products generated from TdT reaction were then employed in a 10 μl final volume of BST–DSN reaction master mix containing Biotin-11-dUTP (B-dUTP) and anchored-oligo-dT (Supplementary Table S3). Nucleotide removal kit (Qiagen) was used to purify the TBD products and the size of TBD products was analyzed on an Agilent DNA Chip 1000 analyzer (Agilent). Reactions were repeated at least three times to check the repeatability of results.

### Hybridization capture

#### On-bead hybridization capture

At first, capture using BST–DSN probes generated from PCR products was examined employing an on-bead hybridization capture procedure as described by Maricic *et al.* (5). Briefly, 800 ng of BST–DSN probes generated from a mix of 10 PCR products were denatured at 98°C for 2 min and immobilized on Dynabeads™ magnetic beads (Thermo Fisher Scientific) at room temperature for 15 min. Immobilized beads were then washed three times with 1× BWT (1 M of NaCl, 5 mM of Tris–Cl, pH8.0, 0.5 mM of ethylenediaminetetraacetic acid (EDTA), pH8.0) and 0.05% of Tween-20 buffer and re-suspended with 500 ng denatured LMPCR products in 1× oligonucleotide buffer, following by incubation at 65°C for 16 h. After washing one time with BWT buffer and two times with 1× Phusion buffer, the captured DNA was released in 1× Phusion buffer by incubation at 95°C for 2 min. Target-specific capture was then validated by two-step PCR (Supplementary Figure S1).

#### In-solution hybridization capture using IDT hybridization and wash kit

In-solution hybridization capture was performed using xGen hybridization and wash kit according to the IDT protocol to examine the sequence-capture ability of TBD probes generated from Panel A/B/C. The NTC_TBD, which is a No Template Control experiment where all steps are included in the absence of input DNA, was used as a negative control in capture. Briefly, LMPCR products (500 ng for the first capture and 200 ng for the second capture) and 7.5 μl of Human Cot DNA were concentrated by 1.8× AMPure XP beads (Beckman Coulter) and re-suspended in 1× hybridization buffer with hybridization enhancer, blocking oligos and probes prior to incubation at specific hybridization temperature for 16 h. The hybrids formed between LMPCR targets and biotinylated probes were immobilized on Streptavidin beads at hybridization temperature for 45 min. Heat-wash was then applied at hybridization temperature with 1× Stringent Wash Buffer for 5 min, following by washing with wash buffer 1, 2 and 3 at room temperature. The captured DNA was then re-suspended in 20 μl Nuclease-Free water. Post-capture LMPCR was performed in a final volume of 50 μl Q5 master mix (NEB) with 1 μmol/l of each Illumina adaptor primer, respectively. Post-capture PCR was performed as description on Supplementary Table S1, following by purification using 1.8× AMPure beads (Beckman Coulter). The purified DNA was then eluted in 20 μl of 1× low-EDTA TE buffer (Quality Biological). Only one round of capture was performed for the original (commercial) probes, while either one or two rounds of capture was performed for TBD probes. The hybridization was performed at 65°C or at 60°C for the original commercial probes, per manufacturer's specifications. The hybridization temperature for TBD probes generated from Panel A was performed at 50°C or 60°C for the first capture, and 60°C for the second capture. For TBD probes generated from Panel B/C, 50°C was used for the hybridization in both first and second capture (Supplementary Figure S2)

#### Two-step PCR validation after capture

Two-step PCR was initially used to validate the presence of discrete DNA targets following target enrichment. The captured DNA was first amplified on CFX Connect^Tm^ real-time PCR machine (Bio-Rad Laboratories) using Illumina adaptor primers in a final volume of 25 μl using Phusion polymerase master mix per the protocol provided on Supplementary Table S1. After amplification using Illumina adaptor primers, DNA was diluted 500× in Nuclease-Free water. A total of 2 μl of diluted DNA was employed as template for target specific PCR Prep, Supplementary Table S1.

#### Illumina MiSeq sequencing and data analysis

The captured DNA was underwent library preparation using NEBNext Ultra II DNA Library Kit for Illumina (NEB). Samples were quality and quantity tested by Agilent Bio-analyzer and KAPA Library Amplification Kit (KAPA Biosystems), and then pooled in a single tube prior to Illumina MiSeq Sequencing at Molecular Biology Core Facility at Dana-Farber Cancer Institute. Human genome hg19 was employed as template for alignment prior to data analysis conducted via the ngsCAT software tool (8) and Picard (http://broadinstitute.github.io/picard/) analysis. The on-target percentage and coverage were analyzed by ngsCAT software, whereas the fold-80-base-penlty was analyzed via Picard. Only mapped reads were included in the analysis, as per ngsCAT requirement. Samtools was used to filter the mapped reads, remove the duplicated reads and perform sorting and indexing prior to ngsCAT analysis. The default ngsCAT parameters were used, except that coverages of 50×, 80×, 100×, 150×, 200×, 400×, 600×, 1000×, 5000×, 10 000×, 20 000× and 50 000× were included using coveragethrs as per ngsCAT capture assessment tool for NGS data.

## RESULTS

### BST–DSN reaction

**Figure 2.**
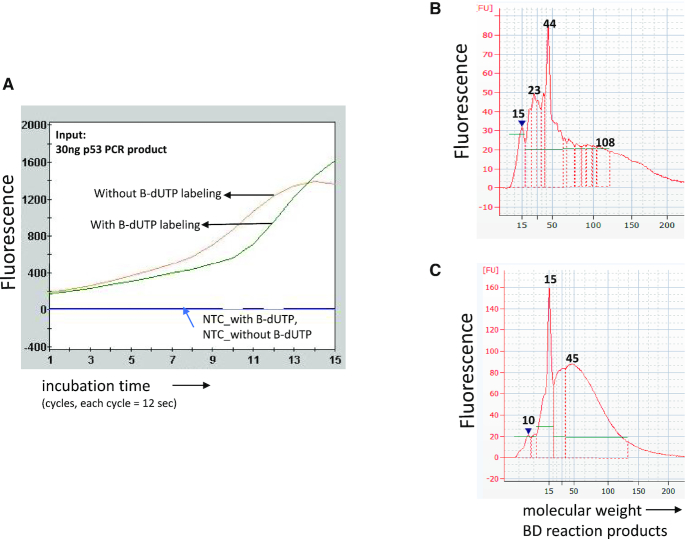
Amplification of dsDNA with/without B-dUTP labeling in BST–DSN reaction. (**A**) A PCR product, p53 exon 8, was used as input in BST–DSN reaction using native nucleotides (dNTPs), or alternatively, dNTPs plus biotinylated dUTP. After amplification, BST–DSN products were purified and the product size was analyzed via electrophoresis on an Agilent Bioanalyzer for native dNTPs (**B**) or for dNTPs plus B-dUTP (**C**). Similar fragment sizes were obtained in the two cases. Under the conditions applied, most BST–DSN products were between 20 and 80 bp while the range of products was ∼15–150 bp.

BST–DSN reaction with dsDNA (PCR products): We first applied BST–DSN reaction (Figure 1) using a single PCR product as input (p53 exon 8, 157 bp amplicon) and the reaction was monitored in real time using a DNA intercalating dye (Figure 2A). Following completion of the reaction, the size range of BST–DSN products was measured via electrophoresis. The BST–DSN reaction, with or without B-dUTP labeling, amplified linearly at the beginning and entered an exponential phase after ∼2.5 min incubation at 65°C. Most BST–DSN products from a single PCR amplicon were in a size range of 20–80 bp (Figure 2A–C). Next, a mix of 10 PCR products, containing varying amounts of 10–200 ng total DNA, was used as input in the presence of B-dUTP. The amplification of the 10 PCR products reached plateau after ∼3 min incubation (Supplementary Figure S3A–D). Moreover, BST–DSN products 20–80 bp size were generated irrespective of DNA input amount under the conditions applied, while the full range of products was 15–150 bp.

BST–DSN reaction with ssDNA (synthetic oligonucleotides): Single stranded oligonucleotides (107–2313 bp long) were used with TdT and followed by BST–DSN reaction to produce amplified B-dUTP -labeled probes. The probe sets tested were a set of 33 target-specific 120 bp long oligonucleotides previously used in our laboratory for hybridization capture and target enrichment prior to sequencing (Panel A, ‘ultramers’ from IDT, 120 bp/probe); the NEBNext™ Direct hotspot cancer panel (Panel B, 190 targets covering 50 genes probes, 107–2313 bp/probe) and the xGen Pan Cancer panel (Panel C, 7816 ultramer probes covering 127 genes, 120 bp/probe). Results showed that 2800, 4500 and 3300 ng of TBD probes were generated from an initial 10, 2.7 and 112 ng of Panels A–C, respectively. The median probe size is 69 bp and the range of probe sizes is 20–120 bp (Supplementary Figure S4).

### Application of biotinylated TBD probes for target-specific capture

To test the application of biotinylated probes toward target-specific capture from amplified genomic DNA, we used the products of TBD reactions as part of DNA sample preparation protocol prior to sequencing. Circulating DNA obtained from normal volunteers was end-repaired, ligated to adaptors and amplified via LMPCR. The LMPCR product was then used for target-specific enrichment using the biotinylated BST–DSN probes for target capture on streptavidin-coated magnetic beads.

#### Target capture using probes generated using a mix of PCR products

As a first test for hybrid capture, biotinylated BST–DSN probes generated from an equimolar mix of 10 PCR products were bound to streptavidin beads and then used to enrich the 10 specific targets from LMPCR product, according to the incubation protocol by Maricic *et al.* (5), Supplementary Figure S1. Following washing steps, 10 specific targets (‘On-Target’) and three non-specific targets (Off-Target) were examined by target-specific PCR. All specific targets showed amplification from bead-bound DNA, while no amplification from the non-specific targets was observed (Supplementary Figure S5).

To examine whether BST–DSN probes capture both wild-type (WT) and mutant alleles from target DNA, BST–DSN capture probes corresponding to a mutation-containing target (NOP14 gene PCR product) were used. These were used for target capture using either HMC WT control LMPCR product or LMPCR product from DNA containing NOP14 mutation with mutation allelic frequency mutant allele frequency (MAF) of ∼81%. After capture, nested PCR followed by Sanger sequencing was applied. An MAF of 0 and 71% were observed using WT and mutation-containing LMPCR products, respectively (Supplementary Figure S6).

#### Target capture using TBD probes generated from Panel A (33-target long-oligonucleotide ‘ultramer’ mix)

TBD probes generated from 33 biotin-labeled ultramers, Panel A, were used in capture reactions from LMPCR products, followed by sequencing. In this workflow, Supplementary Figure S2, biotinylated probes are first hybridized to the target DNA in solution and then bound to beads, as per manufacturer protocol. Either a single or two rounds of target-specific capture was applied to the biotinylated TBD probes. The amounts of TBD probes used as input in the hybridization reaction was varied to assess the impact on capture efficiency, Supplementary Table S4. The captured DNA was analyzed by MiSeq-based sequencing and the capture ability was examined using on-target sequence ratio, coverage and uniformity using the ngsCAT tool (8). The data show that a single round of capture using TBD probes displays slightly inferior on-target percentage and uniformity compared to a single round capture using the original commercial probes, Supplementary Figure S7A. Performing a second capture via TBD probes show that a second capture using TBD probes produces comparable results to a single round capture using the original probes (35–40% on-target ratio for either case). The uniformity was examined via two parameters, the percentage of on-target covered position and the fold-80-base-penalty (the fold of additional sequencing required to bring 80% of target bases to the mean coverage level). Similar conclusions apply to the uniformity using the original probes versus TBD-probes (Supplementary Figure S7B). The analysis of fold-80-base-penalty also showed similar results, 2.55 and 4.75 on the first and second round capture using TBD probes and 6.23 using original probes (Supplementary Figure S7C).

To investigate how GC content and secondary sequence structure affect hybridization capture, the second capture coverage using TBD probes generated from Panel A ultramers, as well the unmodified commercial ultramers were analyzed. The data indicate a similar dependence of coverage and GC content/secondary structure and free energy for TBD probes and the unmodified ultramers. In both cases, increasing coverage correlates with increased GC content and reduced secondary structure/free energy (Supplementary Figure S8).

The ability of TBD probes to recover targets containing low-level mutations was also investigated by applying the same protocol to a DNA sample from a cancer patient. LMPCR product from patient 295 was diluted 20-fold into WT LMPCR product obtained from a normal volunteer, to generate DNA containing panel A target mutations close to mutation allelic frequency MAF∼1%, which is at the detection limits of Miseq sequencing analysis (9). Supplementary Table S5 shows that, most targets anticipated to harbor mutations at the ∼1% level are detected by both ultramer-based capture and TBD probe-based double capture. No mutations at these targets were detected when LMPCR product from normal volunteers was used (not shown).

#### Random pattern of BST–DSN probe generation

To investigate the distribution of DSN cutting positions during TBD probe generation, we also ligated sequencing adaptor to TBD probes and subjected the probes to Miseq sequencing. The first nucleotide in each sequencing read was then assumed to represent a DSN cutting site, since BST synthesis starts from this position. Supplementary Figure S9A and B depict four representative sequences from panel A and indicates that DSN digests at every sequence position, with an ∼1.5- to 1.8-fold preference for G and C versus A and T. This approximately random digestion by DSN enables BST to initiate synthesis at almost every sequence position.

#### Target capture using TBD probes generated from Panel B (190 target commercial oligonucleotide panel)

**Figure 3.**
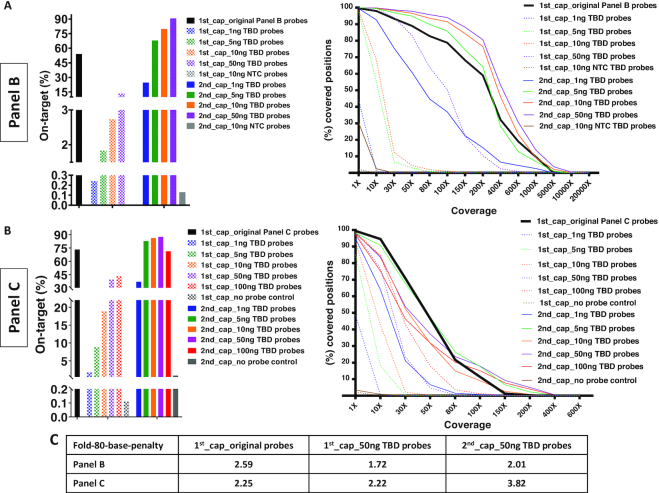
Evaluation of target capture TBD probes generated from Panel B (190 targets) and Panel C (7816 targets). (**A**) Compared to a single round of capture using the original, commercially available probes, using a single round of capture via TBD probes generates inferior on-target ratio. Two rounds of TBD probe capture generate a superior on-target ratio, provided at least 5 ng TBD probes are used as input. (**B**) A similar conclusion as for the on-target ratio applies also to the coverage and (**C**) the fold-80 base penalty, a measure of uniformity.

TBD probes generated using a 190 target cancer-specific panel (NEBNext™ Direct hotspot) was tested next, using the same workflow used for Panel A, and by varying the input 1–50 ng TBD probes during hybridization to LMPCR products. The results (Figure 3A) show that a single round of capture using TBD probes from all conditions has inferior on-target ratio and uniformity performance (1–15%) compared to a single round capture using the original, commercial probes (50%). However, a second round of capture by TBD probes using at least 5 ng TBD probes as input results to a better on-target percentage, 60–90%, as compared to a single round of commercial probes. Similar conclusions apply to the coverage and uniformity, Figure 3A and C. The repeatability of capture was also examined in independent experiments using independently generated TBD probes each time, Supplementary Figure S10A. The standard deviation shown is derived from multiple independent experiments for first and second capture using 50 ng TBD probes generated using Panel B probes.

#### Target capture using TBD probes generated from Panel C (7816 target commercial oligonucleotide panel)

As a next test, TBD probe-based capture generated using a 7816 oligonucleotides comprehensive cancer panel as input was evaluated using the same workflow as for panels A and B. Results depict the same trend as those shown for panel B (Figure 3A and C). A single round of capture using TBD probes at varying input for hybridization to LMPCR product, 1–100 ng, has somewhat inferior on-target ratio and uniformity performance (3–45%) compared to a single round capture using the original, commercial probes (73%). In contrast, the second round of capture using TBD probes revealed superior on-target capture (82–88%) as compared to a single round via the commercial probes (73%). Moreover, a second round of capture using TBD probes showed comparable uniformity to the first capture of the commercial probes (Figure 3B–C). To investigate potential causes for the relatively reduced on-target percentage following first capture using TBD probes, we hypothesized that the fraction of TBD probes with sizes <25 bp (Figure 2) can lead to non-specific hybridization and capture, thereby decreasing the on-target ratio. As a simple test, we repeated the first capture using TBD probes from panel C, at various probe-target hybridization temperatures, 40–60°C, Supplementary Figure S10B and C. The data indicate that performing the capture at 60°C yields better on-target percentage and uniformity. Since smaller TBD probes cannot hybridize at higher temperatures, this result is consistent with the assumption that presence of small-size TBD probes lead to reduction of on-target ratio. Future strategies to avoid non-specific hybridization include the use of higher (∼60°C) hybridization temperatures or application of size-exclusion filtration to TBD probes prior to their use, to eliminate probes smaller than 30–40 bp. Next, the on-target percentage for panels A–C was also assessed by including the target-flanking regions as part of target-specific capture. Minor improvement in target capture was seen in selected cases, Supplementary Table S6 if the flanking regions are assumed to be part of the captured target. At last, the effect of de-duplication of sequencing reads was investigated. The data indicate that removal of duplicated reads leads to a minor (<5%) decrease of the on-target and coverage percentages for both the original Panel C probes (single capture) and the TBD probes (single or double capture), Supplementary Figure S11. The relative amount of on-target and coverage percentages is not significantly affected by de-duplication of reads.

## DISCUSSION

While isothermal amplification reactions using endonucleases for genome-wide (9) or target-specific DNA amplification (10,11) have been described, the action of these enzymes relies on the presence of a DNA recognition sequence on the target DNA and is not random. As such, genomic regions with low-levels of recognition sequences might not amplify effectively. Nucleases, on the other hand, are not dependent on a recognition sequence (6) and can digest homopolymers efficiently (12). Thereby BST-DSN would be expected to amplify and generate probes with good representation of any double stranded DNA template, independent of sequence. Indeed, the data in Supplementary Figure S9 show that DSN digests sequences at almost every position, thus initiating synthesis in a sequence-independent manner. In contrast, CviPII ‘nickase’-based amplification as described by Chan *et al.* (9) requires -CC- for digestion and would not produce random nicking on these sequences (Supplementary Figure S9A, **arrows**). Accordingly, by replacing endonucleases with DSN nuclease in a BST–DSN chain reaction we generate capture probes likely representing all sections of an original sequence. Consistent with this expectation, TBD amplification reactions produce capture probes leading to uniform capture of DNA targets from amplified genomic DNA.

**Figure 4.**
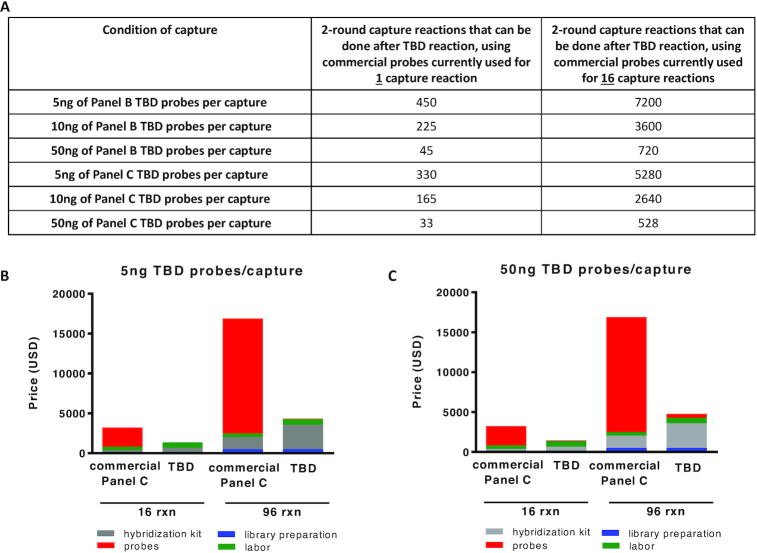
TBD efficiency and resource savings. (**A**) Number of double-capture reactions that can be performed following TBD generated probes, starting from oligonucleotide probes currently used for either one capture or for 16 capture reactions. (**B**) The cost of target enrichment prior to NGS using the original probes of Panel C was compared to the double-capture using 5 ng of TBD probes per round of capture, or (**C**) 50 ng of TBD probes per round of capture. TBD probes largely reduce the cost of target enrichment prior to NGS sequencing for either 16 or for 96 capture reactions. Commercial list prices were used for this comparison.

The data in Figure 4 indicate that by amplifying commercial panels of oligonucleotides prior to performing capture reactions enables major reagent savings. For example, using commercial probes suitable for a single capture reaction as the DNA input in a TBD reaction (Panel B, 190 targets) produces 4500 ng of TBD probes, which is enough for 45–450 double-capture reactions using biotinylated TBD probes (Figure 4A). Also, using commercial probes appropriate for a single capture reaction as the DNA input in a TBD reaction (Panel C, 7186 targets) produces 3300 ng of TBD probes, which is enough for 33–330 double-capture reactions using biotinylated TBD probes (Figure 4A). TBD reactions are complete in minutes, while the overall process with purifications is <2 h. Moreover, a two-round capture using TBD probes produces excellent (>80%) on-target ratio and uniformity (fold-80-base penalty). In effect, the reagent cost for target capture is diminished by following the present approach, albeit at the cost of introducing an additional capture step. This additional step increases labor cost, but as Figure 4B and C shows, the overall cost of sample preparation is reduced a lot. Reducing cost of sample preparation reagents lowers the overall cost of targeted re-sequencing. Additional reductions in overall re-sequencing cost can be achieved via mutation enrichment approaches which reduce the number of WT molecules that needs be sequenced (7,13). For example, PCR amplification using WT DNA-suppression approaches (14,15) have been shown to boost the mutant allelic fraction and reduce the amount of sequencing required to call mutations (16,17), in addition to increasing mutation detection threshold in conventional sequencing applications (18).

Compared to an alternative way for amplification of commercial capture probes by synthesizing probes with two universal regions, then amplifying the universal regions with biotinylated primers, the TBD method has the advantage of amplifying any pre-existing set of probes without requiring sequence information and without presence/absence of universal regions. Most manufacturers currently do not provide information on universal regions hence TBD enables small laboratories to reduce cost on expensive capture probes irrespective of commercial format. Further, including universal regions in the probes may promote probe self-hybridization during capture unless a new ‘blocker’ oligonucleotide is used to prevent this. A disadvantage of the TBD method is that, under the current capture protocol, two sequential capture reactions are needed instead of one to achieve high ‘on-target’ fraction.

In summary, we presented a simple approach to amplify panels of double-stranded DNA or long oligonucleotide probes used for target enrichment prior to sequencing, using a novel nuclease-polymerase isothermal chain reaction. This approach enables an increase in efficiency and significant reduction in cost of reagents used for sample preparation in targeted re-sequencing applications employing hybrid capture and should be broadly applicable to both human, non-human and ancient DNA applications.

## DATA AVAILABILITY

The authors declare that all data supporting the findings of this study are available within the paper and its Supplementary Data.

## Supplementary Material

gkz870_Supplemental_File
