# Supplementary material for: A nuclease-polymerase chain reaction enables amplification of probes used for capture-based DNA target enrichment
Source: Nucleic Acids Res. 2019 Oct 10;47(22):e147. doi: 10.1093/nar/gkz870 (PMC6902007; doi:10.1093/nar/gkz870)
Supplement: gkz870_Supplemental_File [file gkz870_supplemental_file.pdf]

Supplementary Figure 1.

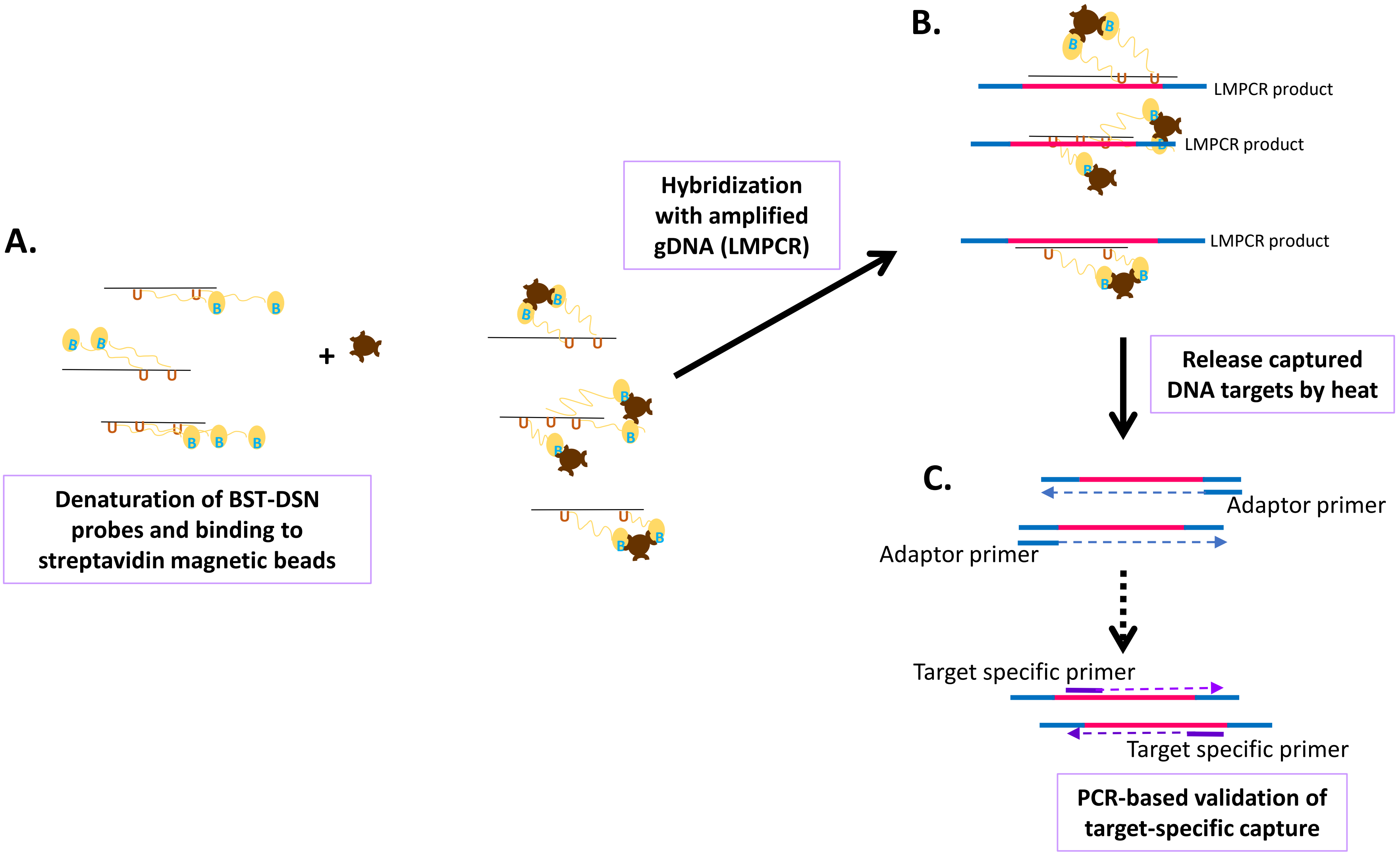

Supplementary Figure 1. Target-specific capture using BST-DSN probes pre-attached to streptavidin beads. (A) biotinylated BST-DSN probes were first immobilized on streptavidin beads. (B) Hybridization of LMPCR products at 65C/60C for 16 hours was performed with labeled beads, followed by washing steps. (C) Captured DNA was released from beads by heating at 98C for 2 min and PCR was performed to validate the target-specific capture.

Supplementary Figure 2.

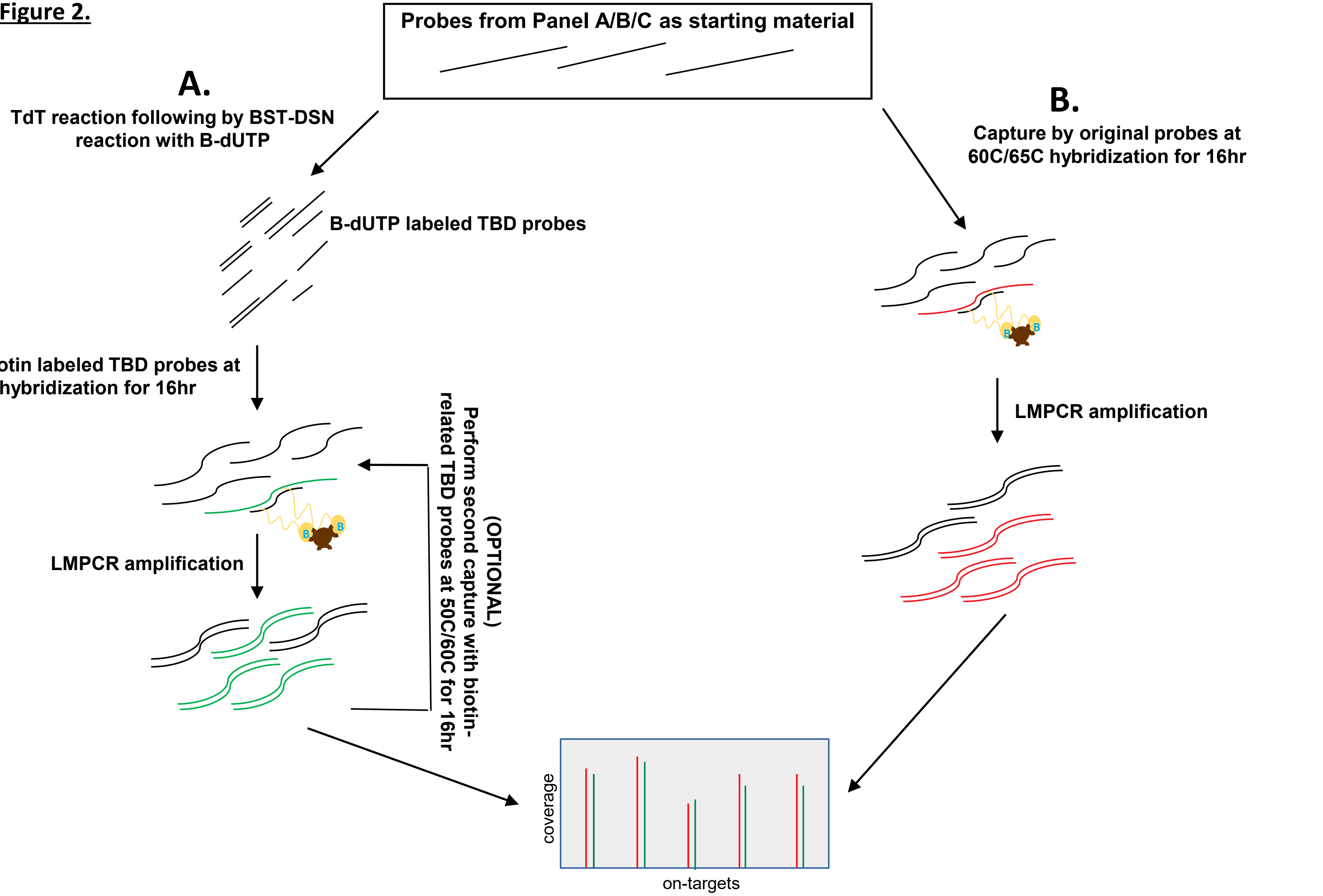

Supplementary Figure 2. Workflow applied for hybridization capture prior to MiSeq sequencing (A) The biotin-labeled commercial oligonucleotide probes from Panels A, B and C were used as starting material for TBD reaction using B-dUTP to generate biotin-labeled TBD probes. These TBD probes were then used to capture specific targets from LMPCR product on streptavidin beads. Following LMPCR of the bead-captured DNA, the product was either sequenced directly or subjected to a second capture and then sequenced. (B) For comparison to TBD-probe based capture, the original biotin-labeled commercial probes were tested in the same protocol, using a single round of capture was performed by original probes and followed by LMPCR amplification. The comparison of on-target captured ability between TBD probes and original probes was then validated by MiSeq sequencing.

Supplementary Figure 3.

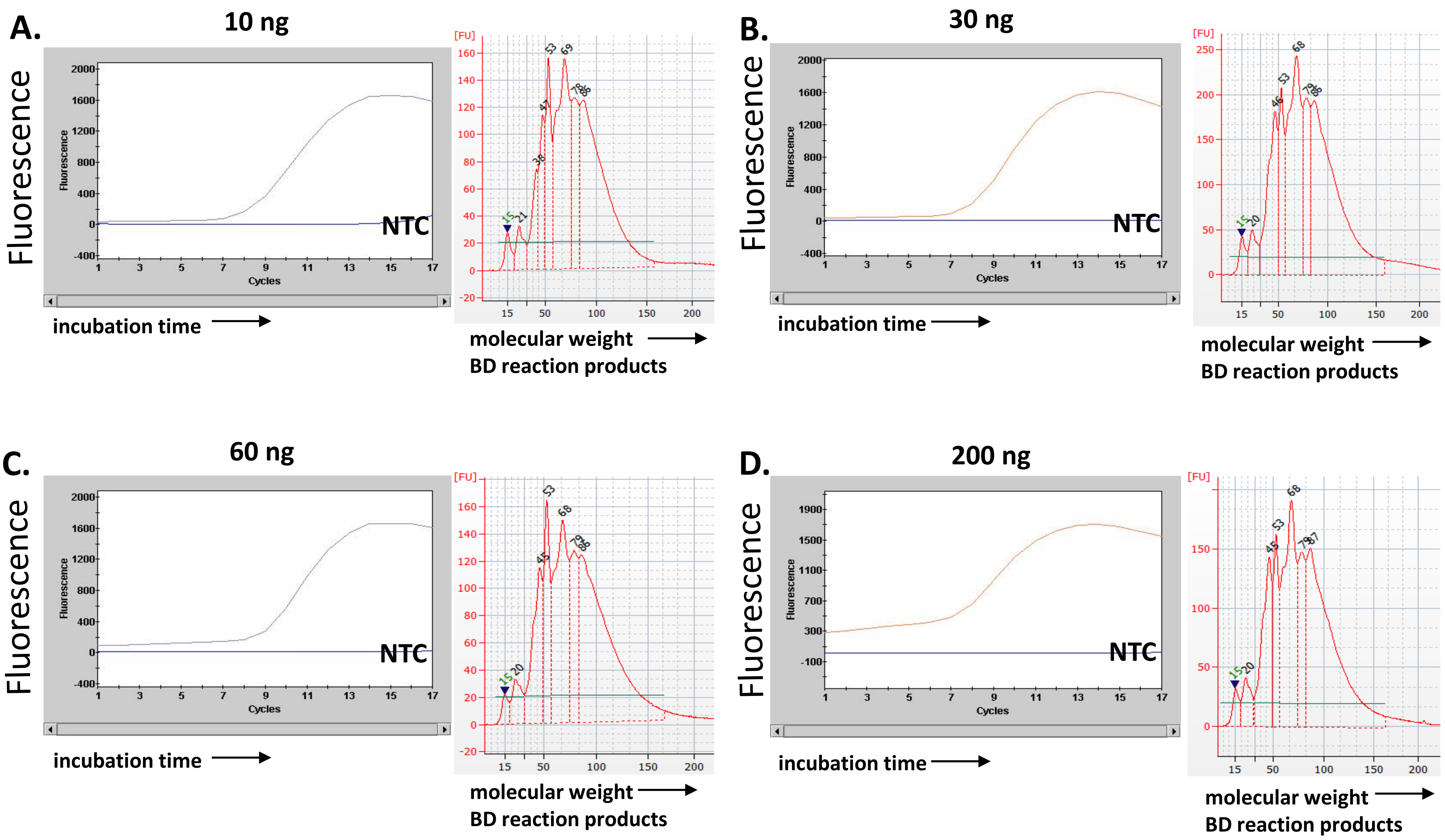

Supplementary Figure 3. Amplification of dsDNA via BST-DSN reaction with concomitant B-dUTP labeling (A) 10ng, (B) 30ng, (C) 60ng or (D) 200ng using a 10 PCR product mix was used as total DNA input in BST-DSN reaction with B-dUTP. Following amplification, BST-DSN product size was analyzed via electrophoresis on an Agilent Bioanalyzer. Under the conditions applied, most BST-DSN products were between 20 and 80 bp while the full range of products was 15-150 bp.

Supplementary Figure 4.

A.

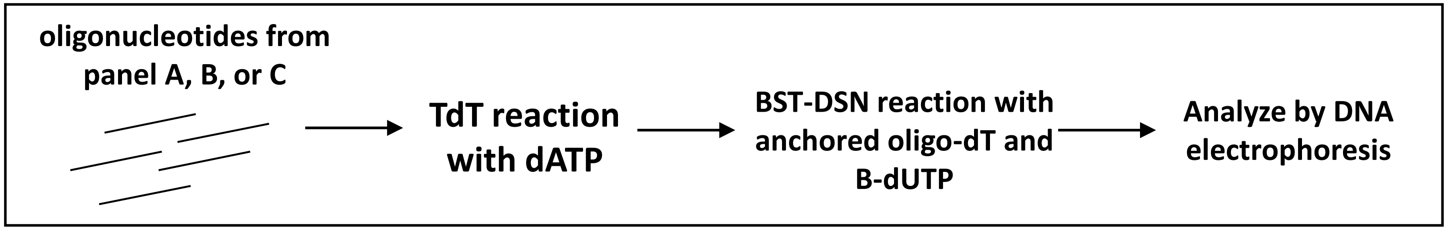

B.

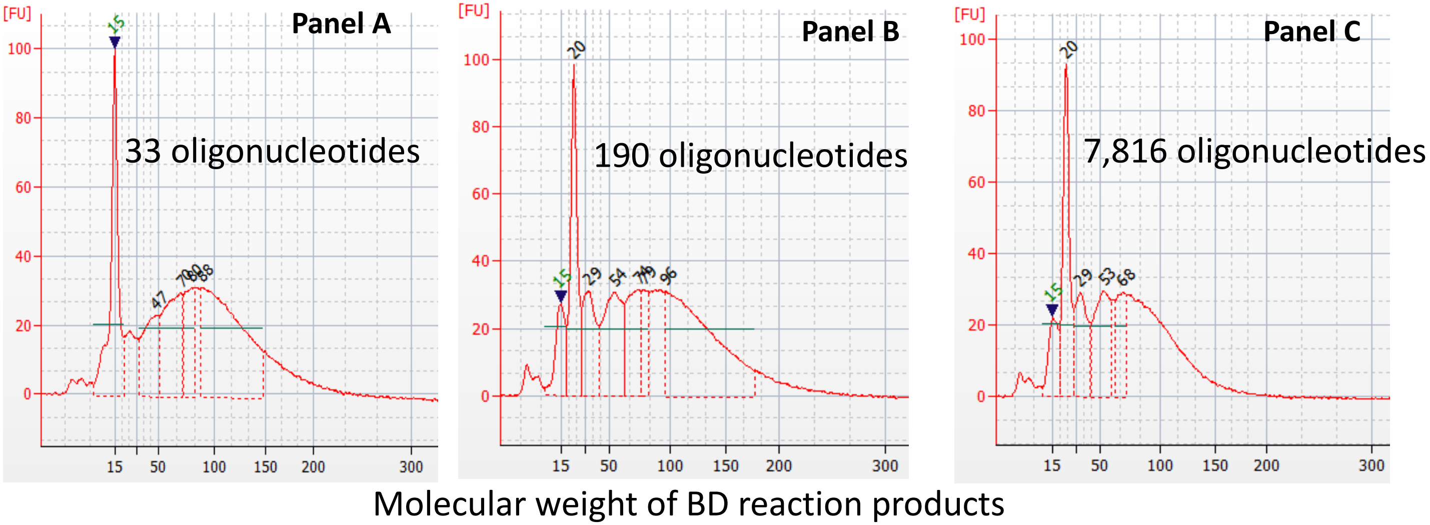

C.

Name	Original probes used in TdT reaction with B-dUTP	Total amount after purification
Panel A	10 ng	2800 ng
Panel B	2.7 ng	4500 ng
Panel C	112ng	3300 ng

Supplementary Figure 4. Amplification of Panel A, B and C by TdT-BST-DSN (TBD) reaction with concomitant B-dUTP labeling (A) workflow used for generating TBD probes from long oligonucleotides in Panels A, B or C. (B) size of B-dUTP labeled TBD products as examined by DNA electrophoresis on an Agilent Bioanalyzer. Product sizes were approximately 20-120 bp. (C.) amount of TBD probes generated from original probes of Panel A, B and C.

Supplementary Figure 5.

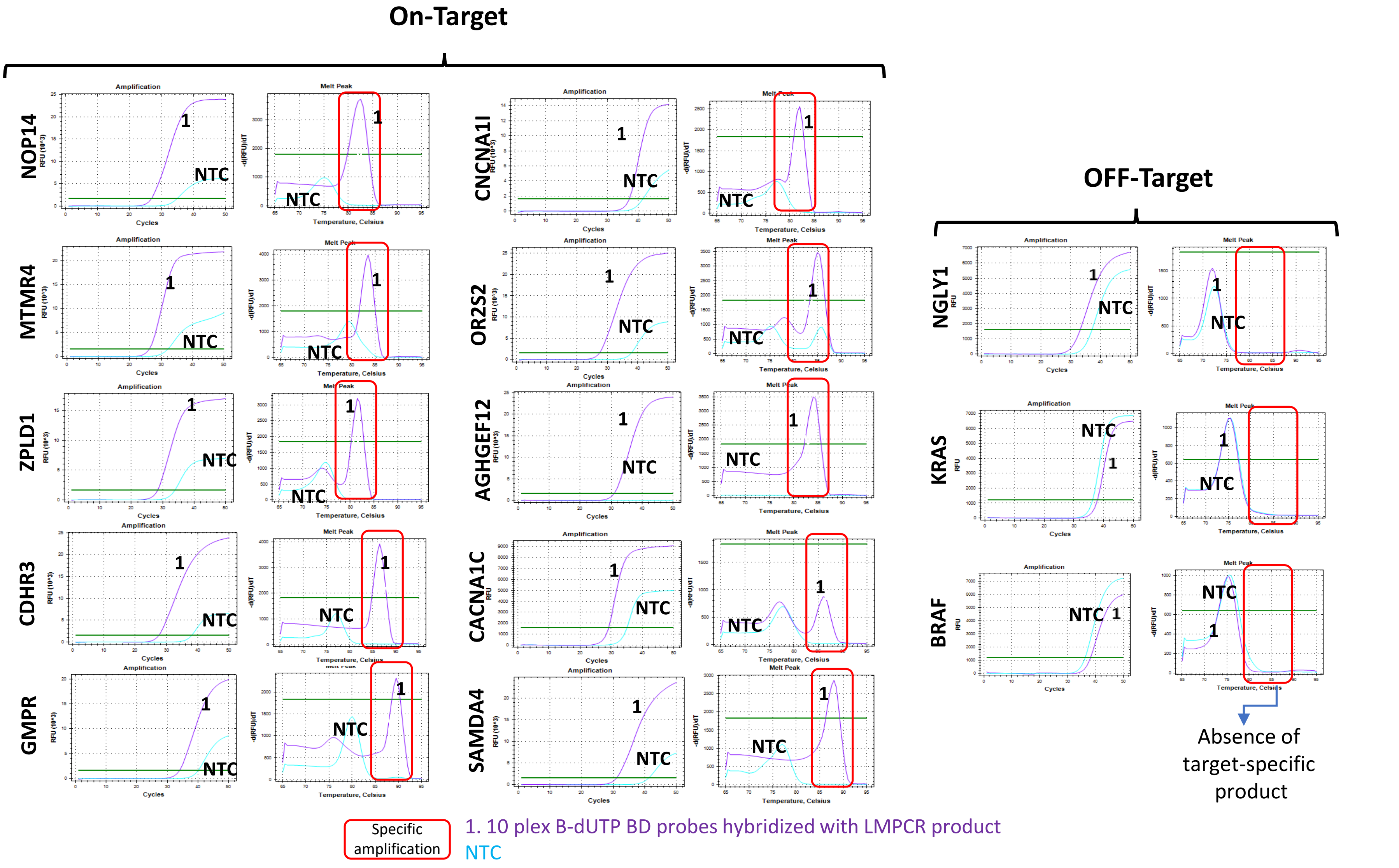

Supplementary Figure 5. Using PCR to validate the target-specific capture from B-dUTP labeled BST-DSN probes generated from a mix of 10 PCR products. 800 ng of BST-DSN probes generated from a mix of 10 PCR products covering biologically relevant DNA targets were applied for capturing DNA from LMPCR products, followed by amplification of the captured DNA. The specificity of capture was verified by target-specific PCR and melting analysis for the 10 specific targets, as compared to off-target PCR applied for randomly chosen targets. All ten targets were specifically amplified from captured DNA, while no amplification from off-target DNA was observed, based on melting curve analysis.

Supplementary Figure 6.

A.

Original 295 library for hybridization

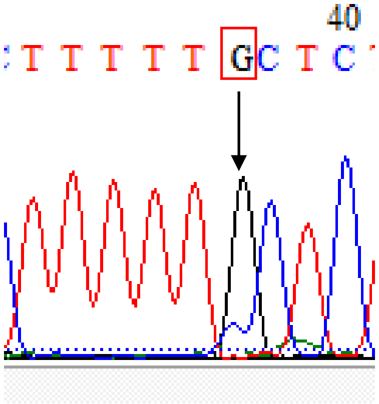

G=81%
C=19%

B.

Captured product:
NOP14 BST-DSN probes hybridized with 295 library

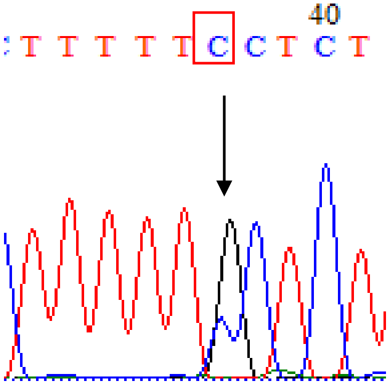

G=71%
C=29%

C.

Captured product:
NOP14 BST-DSN probes hybridized with HMC library

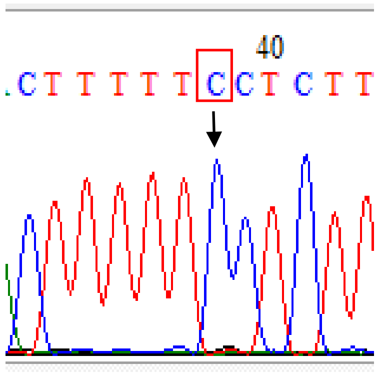

C=100%

WT allele: C
Mutant allele: G

Supplementary Figure 6. Examination of mutant and WT target capture using BST-DSN probes. BST-DSN probes generated from WT NOP14 PCR product were applied for capturing mutated and WT alleles from cancer patient 295 LMPCR product and HMC LMPCR product, following by amplification of the captured DNA. Sanger sequencing was used to verify the mutation allelic frequency MAF of the captured DNA. (A) 81% mutant allele (G) is detected prior to capture from cancer patient DNA (295). (B) 71% mutant allele (G) is detected following capture; (C) 100% WT allele (C) is detected using capture of the same WT NOP14 PCR product hybridized with HMC LMPCR library.

Supplementary Figure 7.

A.

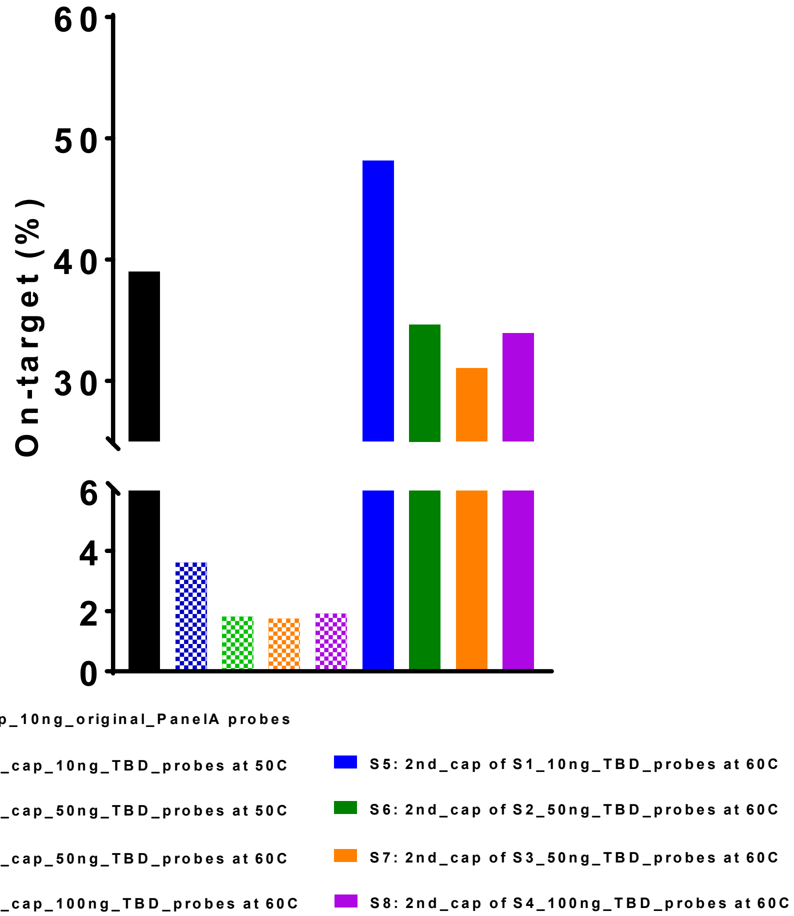

B.

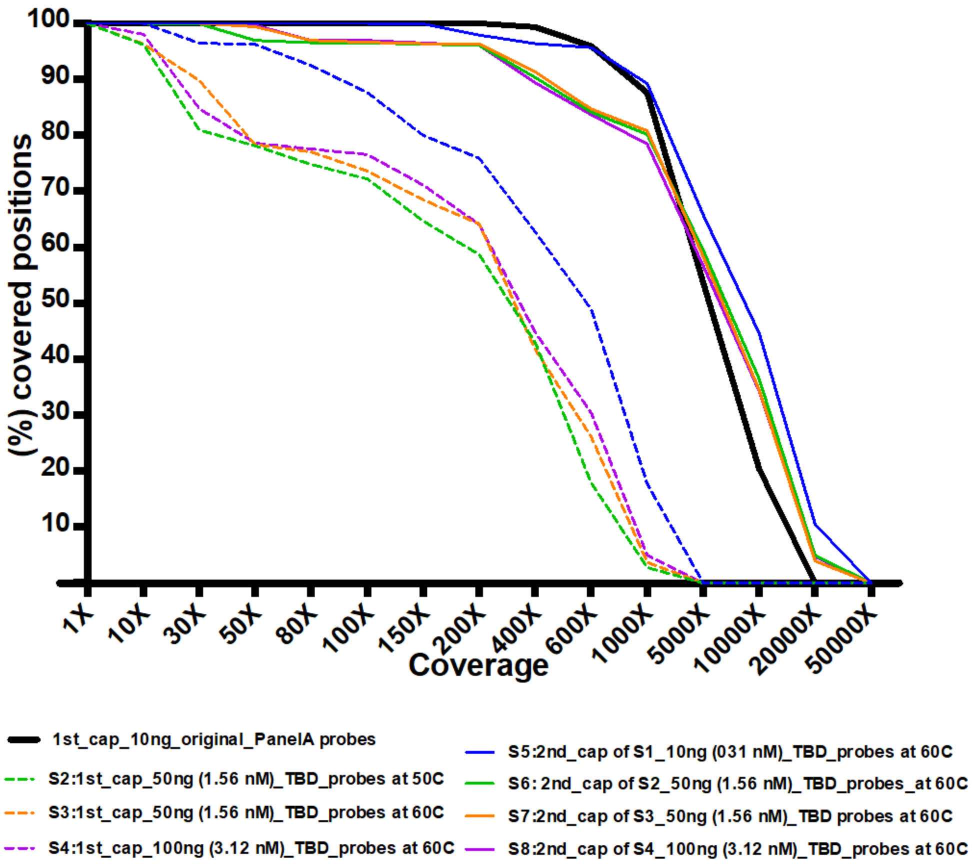

C.

	1st_cap_original Panel B probes	1st_cap_50ng TBD probes	2nd_cap_50ng TBD probes
**Fold-80-base-penalty**	6.23	2.55	4.75

Supplementary Figure 7. Comparison of target capture using BST-DSN probes vs. Panel A commercial capture probes (33) ultramer biotinylated oligonucleotides. (A) A 1st round capture using BST-DSN probes shows inferior on-target percentage (15-20%) as compared to capture using the original probes (40%). A 2nd round capture resulted to similar on-target percentage with a single capture using commercial probes, irrespective of capture probe input, 10-100ng (0.31-3.12 nM). (B) Compared to the 1st capture using commercial probes, the 2nd round of capture of BD probes shows comparable coverage and (C) uniformity with 2nd round capture of BD probes.

Supplementary Figure 8.

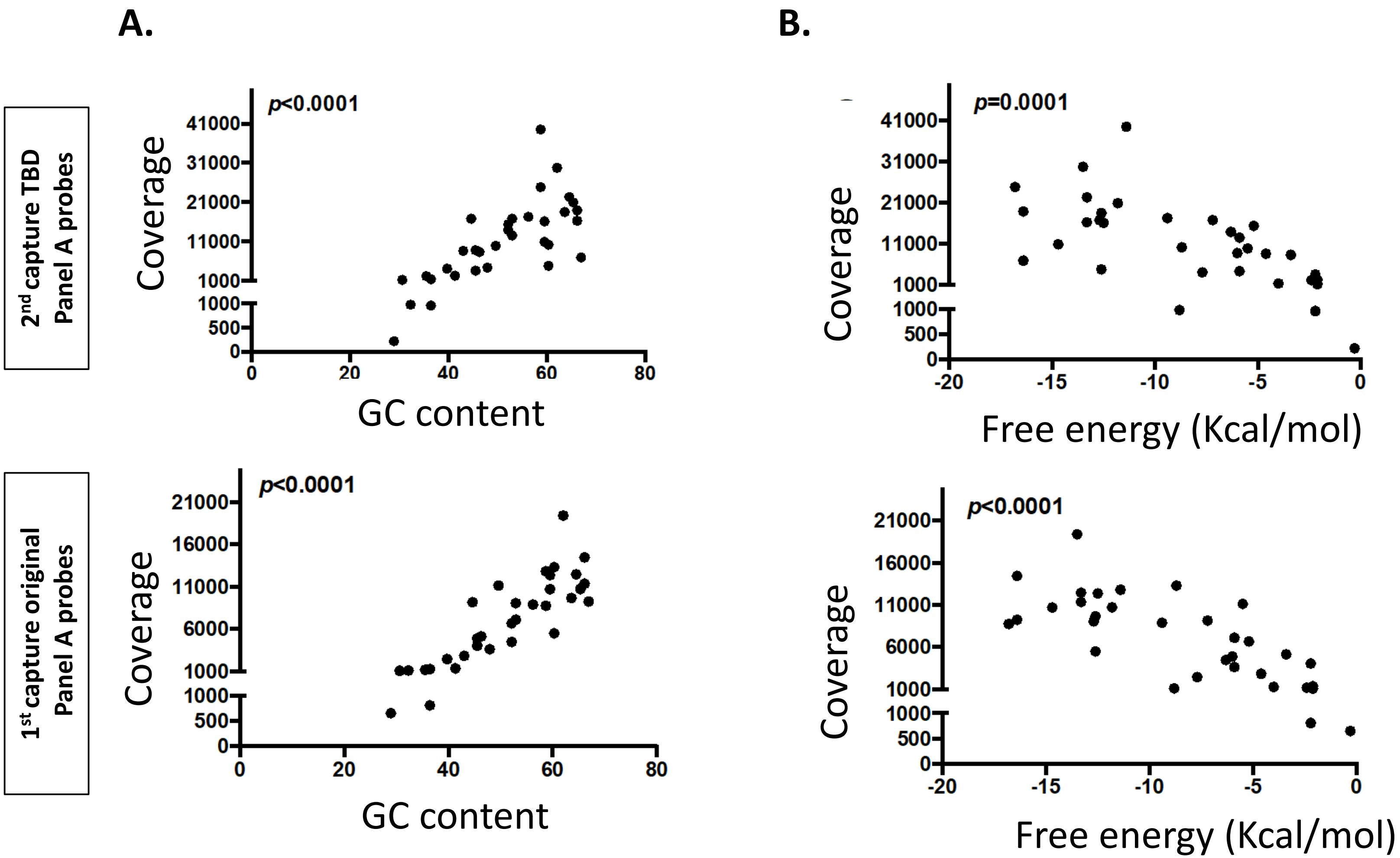

Supplementary Figure 8. Correlation between captured DNA coverage with GC content and free energy, panel A capture probes. A Similar correlation was found between coverage and GC content/secondary structure and free energy for both TBD probes and original ultramers. Kinefold were applied to analyze the free energy. Statistical analyses were performed with PRISM 6 software (GraphPad).

Supplementary Figure 9.

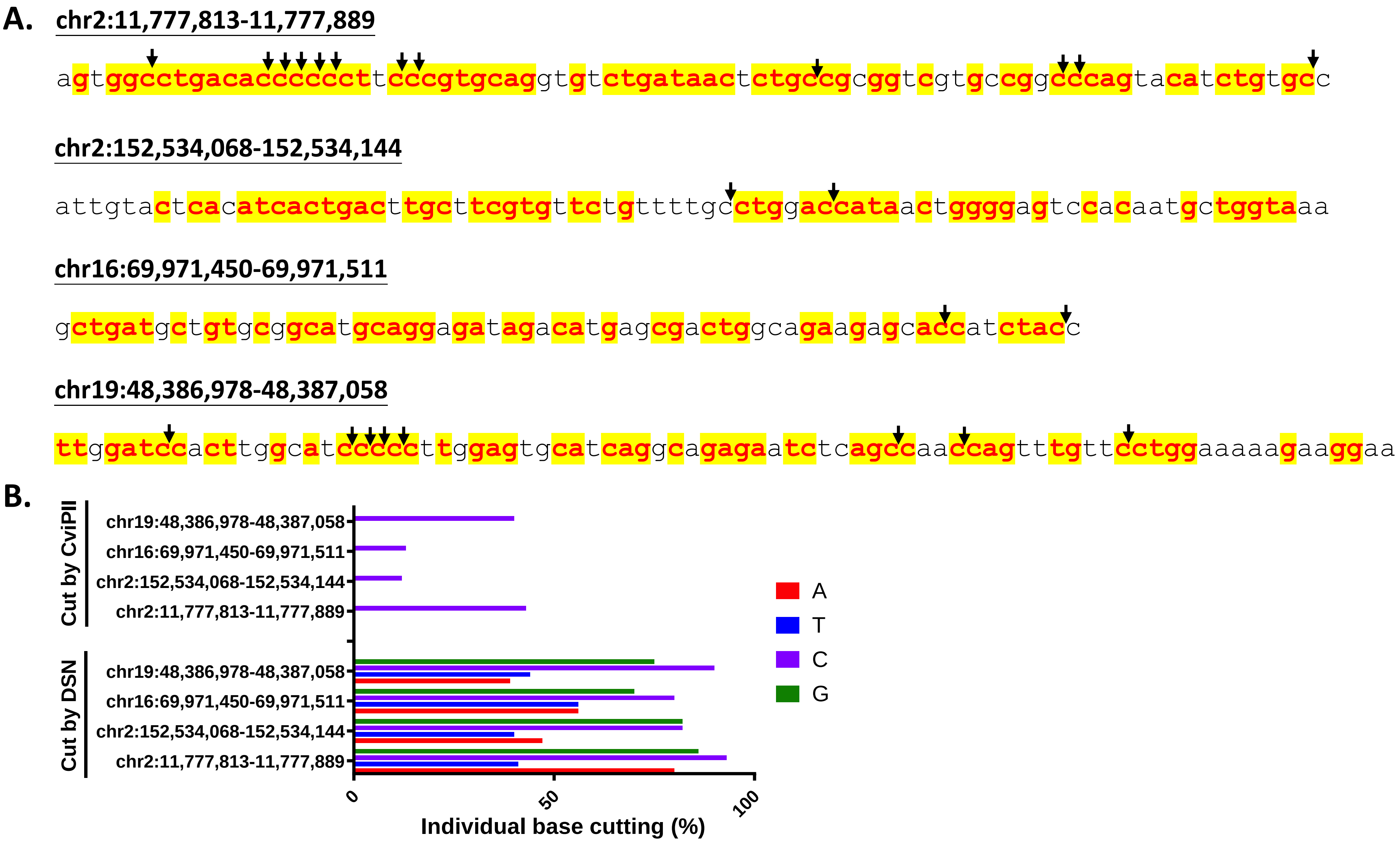

Supplementary Figure 9. DSN digestion points on DNA during a BST-DSN reaction. (A) DSN cutting sites were inferred from MiSeq sequencing data, by examining the starting position of individual sequencing reads on four representative regions. The sequence positions noted in red and highlighted in yellow represent DSN cutting sites at four representative regions of TBD probes generated from Panel A. The arrows indicate the positions where an alternative enzyme (CviPII 'nickase') would be expected to digest the same sequences. (B) Percentage of cutting by DSN and CviPll at individual bases in the four represented sequences from A.

Supplementary Figure 10.

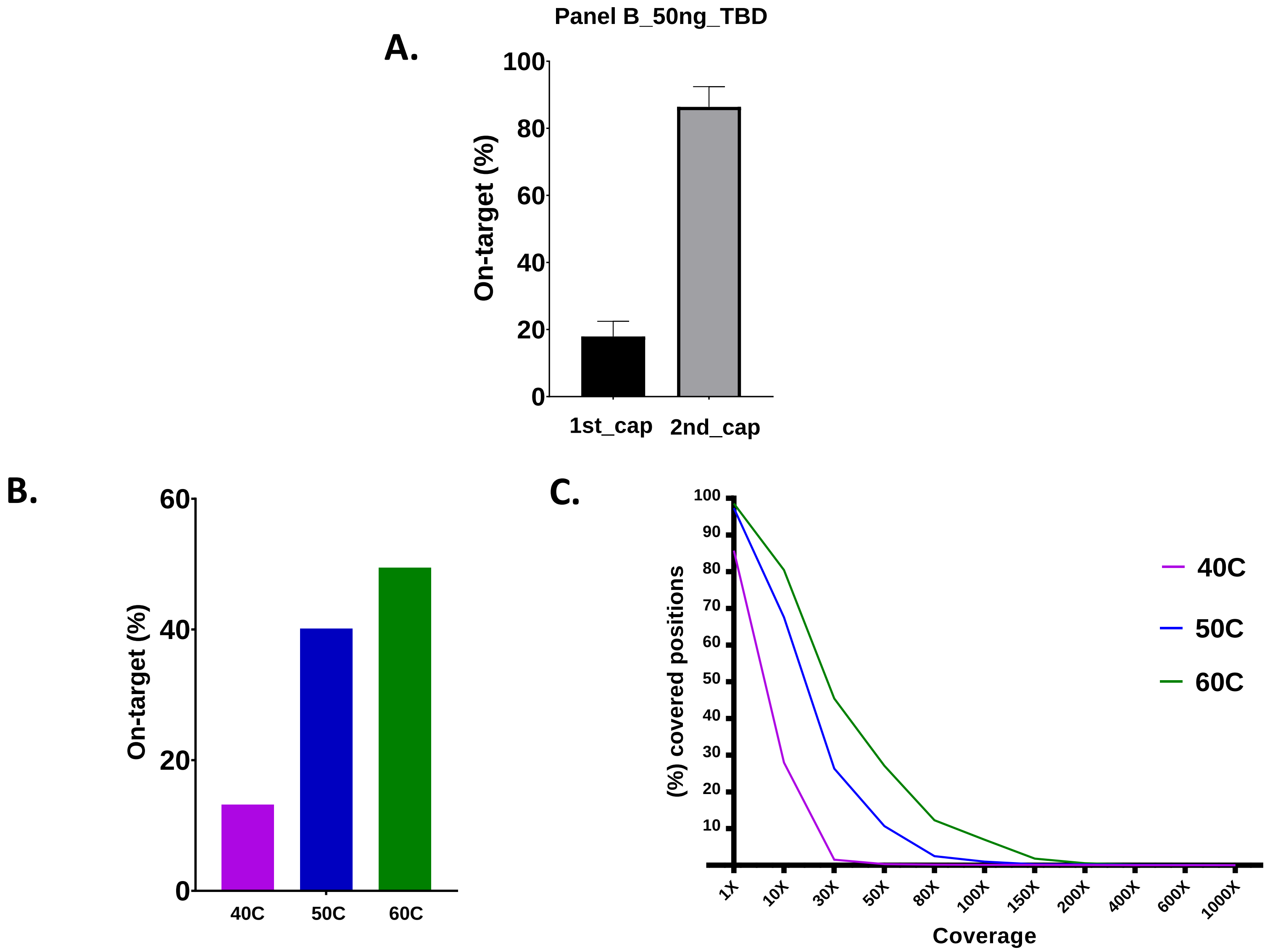

Supplementary Figure 10. Reproducibility of capture and the effect of hybridization temperature of TBD probes. (A) Consistency of on-target percentage obtained in replicate reactions starting from new TBD reactions each time. The standard deviation shown is derived from multiple independent experiments for 1st and 2nd capture using 50ng TBD probes generated from Panel B original probes. (B) On-target percentage and (C) the percentage of coverages were examined using hybridization temperature 40°C, 50°C and 60°C with 100ng of TBD probes generated from Panel C. Increasing the hybridization temperature increased the on-target and coverage percentage for the 1st capture.

Supplementary Figure 11.

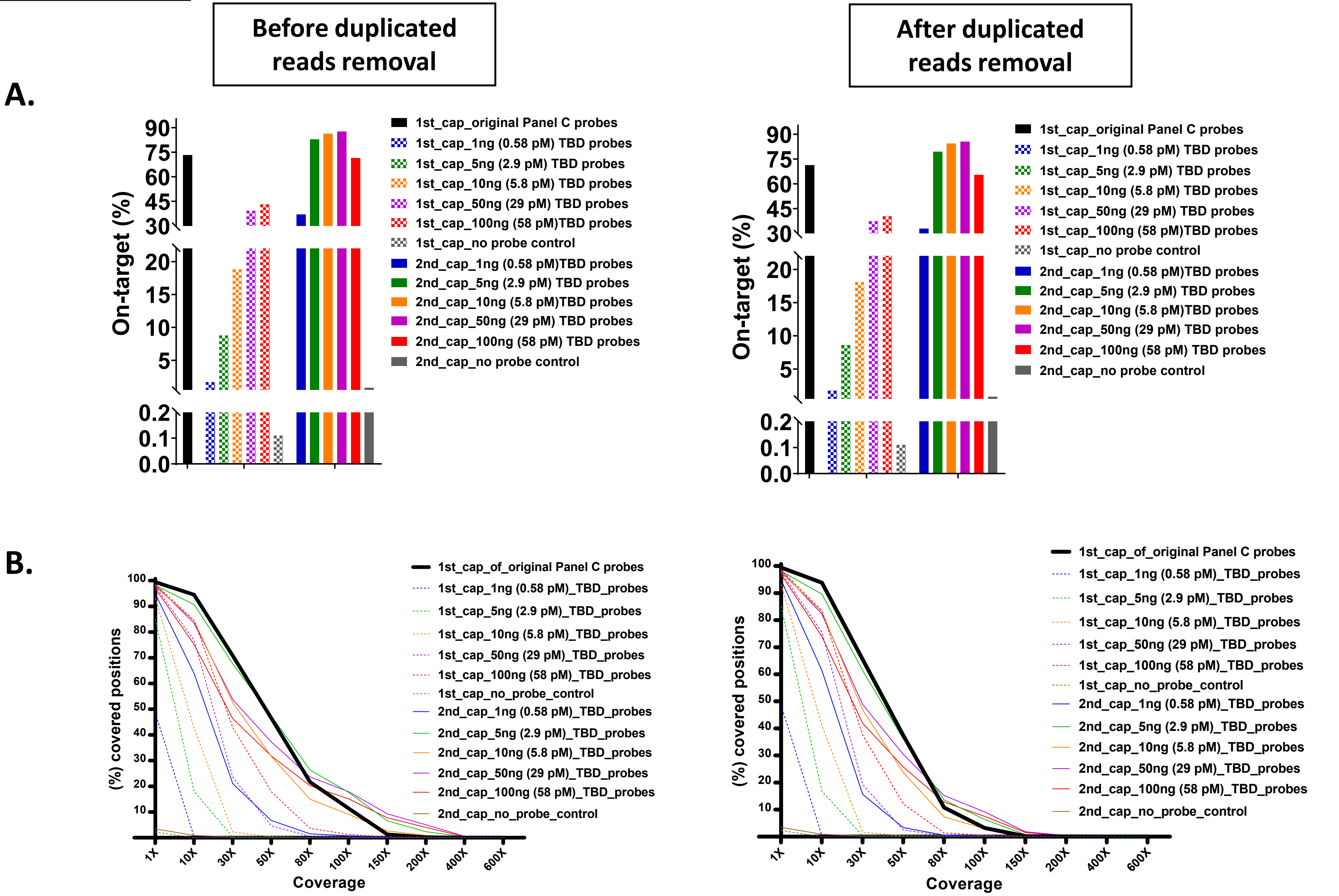

Supplementary Figure 11. Comparison of the capture efficiency before and after duplicated reads removal for probe Panel C. (A) On-target percentage and (B) coverage percentage were examined before and after removal of duplicated reads. A small (<5%) reduction in on-target and coverage percentages was observed for both the original probes undergoing single capture and TBD probes undergoing one or two captures.

Supplementary Table 1. PCR Protocols

PCR reaction for target specific amplification from human genome

Reaction setup	25 µl Rxn (µl)	Final concentration/amount	Thermo cycling
5 X Phusion HiFi buffer	5	1 X	98C 2 min
dNTP (10 mM each)	2	0.8 mM	98C 10 sec
10 X LCGreen	2	0.8 X	65C 30 sec 40 cycles
10 uM primer (F1+R1) from supplementary Table 2	0.5	200 nM	72C 30 sec
Phusion polymerase	0.2	0.2 U	72C 5 min
5 ng Human male genomic DNA	1	5 ng	Melting (optional)
dH_2O	14.3		

Post-capture PCR for LMPCR products

Reaction setup	50 µl Rxn (µl)	Final concentration/amount	Thermo cycling
Q5 master mix	25	1X	98C 30 sec
Illumina adaptor primer (F1+R1) (10 uM)	5	1 uM	98C 15 sec
$MgCl_2$ (25 mM)	1	0.5 mM	65C 30 sec 14 cycles
dH_2O	19		72C 30 sec
			72C 1 min
			4C Hold

Two-step PCR

a.) LMPCR

Reaction setup	25 µl Rxn (µl)	Final concentration/amount	Thermo cycling
5 X Phusion HiFi buffer	5	1 X	98C 2 min
dNTP (10 mM each)	2	0.8 mM	98C 10 sec
10 X LCGreen	2	0.8 X	60C 30 sec 15 cycles
Illumina adaptor primer (F1+R1) (10 uM)	0.5	200 nM	72C 30 sec
Phusion polymerase	0.2	0.2 U	72C 5 min
Captured DNA	10		Melting (optional)
dH_2O	4.3		

b.) Target specific PCR

Reaction setup	25 µl Rxn (µl)	Final concentration/amount	Thermo cycling
5 X Phusion HiFi buffer	5	1 X	98C 2 min
dNTP (10 mM each)	2	0.8 mM	98C 10 sec
10 X LCGreen	2	0.8 X	65C 30 sec 40 cycles
10 uM specific primer (F1+R1) from supplementary Table 2	0.5	200 nM	72C 30 sec
Phusion polymerase	0.2	0.2 U	72C 5 min
1:500 diluted product from LMPCR	1		Melting (optional)
dH_2O	14.3		

Supplementary Table 2: Primers for PCR reactions

Primer ID	Sequence	Functions
NOP14-F1<br>NOP14-R1	GCAGATGTGAGGTAATGTCCAG<br>TGCGGAAAGAAAGCGGAAAG	specific target for 10 plex capture
MTMR4-F1<br>MTMR4-R1	TGCTTCTGAAAGGCAGTCTTC<br>AGCGTTAATGATGATGAAGATAATTTG	specific target for 10 plex capture
ZPLD1-F1<br>ZPLD1-R1	CTGTGAGAGAGAACAATGGC<br>AGCCATTCACATAAATGACTCCT	specific target for 10 plex capture
CDHR3-F1<br>CDHR3-R1	GGTCTGGGTTCCAGAAATGAG<br>AGCATCTCAGTGATTGACAGG	specific target for 10 plex capture
GMPR-F1<br>GMPR-R1	GACGGAAGCTCAAGCTCTTC<br>CCCATCTCCGTAAGAAAAGCA	specific target for 10 plex capture
CACNA1I-F1<br>CACNA1I-R1	CCATTGTGCTACTGTCAGTCAT<br>CCCGTTCCCTGTCCCAC	specific target for 10 plex capture
OR2S2-F1<br>OR2S2-R1	GGATGAGTTTGTCTGAAAGATCCT<br>AGGGGAGGAAAAAGGTCTTC	specific target for 10 plex capture
ARHGEF12-F1<br>ARHGEF12-R1	TGTCTTTTCCTTTCTCTGTTTTCTCT<br>ACTGTACGAAGACTGGATTGTC	specific target for 10 plex capture
CACNA1C-F1<br>CACNA1C-R1	TGGCTTTGCAGGTCGAAGG<br>GTGAGCTACCTGATGATGAACC	specific target for 10 plex capture
SAMD4A-F1<br>SAMD4A-R1	GCCACTTCGTTAGAAGACCG<br>TGGTGATAGTAGTGTGTCTGTCC	specific target for 10 plex capture
NGLY1-F1<br>NGLY1-R1	GCTGTATCAGATCGCAATTTCC<br>CATCTTTTGCTTATATTTCCTGGAAGT	non-specific target for 10 plex capture
KRAS-F1<br>KRAS-R1	ATTTCAGTGTTACTTACCTGTCTTG<br>ACAGGCTCAGGACTTAGCAA	non-specific target for 10 plex capture
BRAF-F1<br>BRAF-R1	ACCACATTACATACTTACCATGCC<br>TGGGCAGATTACAGTGGGAC	non-specific target for 10 plex capture
p53-Ex8-F1<br>p53-Ex8-R1	GAACAGCTTTGAGGTGGGTGTTT<br>TGGTCTCCTCCACCGCTTC	Single target PCR
Illumina adaptor primer-F1<br>Illumina adaptor primer-R1	AC ACT CTT TCC CTA CAC GAC CT CTT CCG ATCT<br>GACTGGAGTTCAGACGTGTCTCTTCCGATCT	LMPCR

Supplementary Table 3. TdT and BST-DSN protocols

BST-DSN reaction WITHOUT B-dUTP labeling

Reaction setup	10 µl Rxn (µl)	Final concentration/amount	Incubation		
10 X BST buffer	1	1 X	65C	6 sec (OFF)	180 sec
10 X LCGreen	1	1 X		6 sec (ON)	
dNTP (10 mM each)	1	1 mM	95C	2 min	
$MgCl_2$ (25 mM)	1.2	3 mM		Melting (Optional)	
BST polymerase (8 U/ul)	1	8 U			
DSN enzyme (1 U/ul)	0.2	0.2 U			
DNA (30 ng)	1	30 ng			
dH_2O	3.6				

BST-DSN reaction WITH B-dUTP labeling

Reaction setup	10 µl Rxn (µl)	Final concentration/amount	Incubation		
10 X BST buffer	1	1 X	65C	6 sec (OFF)	204 sec
10 X LCGreen	1	1 X		6 sec (ON)	
dNTP (10 mM each)	1	1 mM	95C	2 min	
$MgCl_2$ (25 mM)	1.2	3 mM		Melting (Optional)	
Biotin-11-dUTP (1 mM)	1.6	0.16 mM			
BST polymerase (8 U/ul)	1	8 U			
DSN enzyme (1 U/ul)	0.2	0.2 U			
DNA (10 ng, 30 ng, 60 ng or 200 ng)	1	10 ng, 30 ng, 60 ng or 200 ng			
dH_2O	3.6				

TdT reaction

Reaction setup	10 µl Rxn (µl)	Final concentration/amount	Incubation	
TdT enzyme (20 U/ul)	0.1	2U	37C	30min
10 X TdT buffer	1	1 X	75C	20 min
2.5 mM $COCl_2$	1	0.25 mM	4C	Hold
dATP (10 mM)	0.5	0.5 mM		
Panel A/B/C	2.5	10 ng Panel A/2.7 ng Panel B/112.36 ng Panel C		
dH_2O	5			

BST-DSN reaction after TdT reaction

Reaction setup	10 µl Rxn (µl)	Final concentration/amount	Incubation		
10 X BST buffer	1	1 X	60C	6 sec (OFF)	*incubation time
10 X LCGreen	1	1 X		6 sec (ON)	
dNTP (10 mM each)	1	1 mM	95C	2 min	
$MgCl_2$ (25 mM)	1.2	3 mM		Melting (Optional)	
Biotin-11-dUTP (1 mM)	1.6	0.16 mM			
Anchored-oligo-dT (500 ng/ul)	0.2	100 ng			
BST polymerase (8 U/ul)	1	8 U			
DSN enzyme (1 U/ul)	0.2	0.2 U			
Product from TdT reaction	2				
dH_2O	1				

*** 420 sec for Panel A and B, 204 sec for Panel C**

Supplementary Table 4. Concentration of TBD probes used in capture

**Panel A (33 targets)**	Concentration (total)	Concentration (each)
10ng_TBD_probes	10.3 nM	0.31 nM
50ng_TBD_probes	51.5 nM	1.56 nM
100ng_TBD_probes	103 nM	3.12 nM

**Panel B (190 targets)**	Concentration (total)	Concentration (each)
1ng_TBD_probes	4.53 nM	0.02 nM
5ng_TBD_probes	22.66 nM	0.12 nM
10ng_TBD_probes	45.32 nM	0.24 nM
50ng_TBD_probes	226.59 nM	1.19 nM

**Panel C (7816 targets)**	Concentration (total)	Concentration (each)
1ng_TBD_probes	4.53 nM	0.58 pM
5ng_TBD_probes	22.66 nM	2.9 pM
10ng_TBD_probes	45.32 nM	5.8 pM
50ng_TBD_probes	226.59 nM	29 pM
100ng_TBD_probes	453.19 nM	58 pM

Supplementary Table 5. Application of original ultramer probes, or ultramer-derived TBD probes to capture DNA targets with low-level mutations

DNA target	position	mutation	Panel A ultramer probe capture MAF	Panel A TBD probe double capture MAF
MAP10	chr1:232942818	C>G	0.26% *	1.18%
RBM8A	chr1:145507691	G>A	1.82%	2.64%
EVI5	chr1:93029240	T>C	1.18%	1.78%
NPHS1	chr19:36322234	G>C	1.50%	0.33% *
SPACA4	chr19:49110373	C>G	0.22% *	1.22%
RBM8A	chr1:145507691	G>A	1.82%	2.64%
GREB1	chr2:11777848	G>T	1.12%	0.51% *

* Value near of below noise threshold of ~0.5%

Supplementary Table 6. Comparison of the on-target captured percentage using the probe regions with/without 300 bp flanking region

Panel A

samples	on-target (%)	on-target (%) with 300bp flanking region
1st capture with 10ng TBD probes	3.6	4.5
2nd capture with 10ng TBD probes	48.1	60.1
1st capture with Panel A original probes	38.9	46.8

Panel B

samples	on-target (%)	on-target (%) with 300bp flanking region
1st capture with 50ng TBD probes	13.4	13.5
2nd capture with 50ng TBD probes	90.7	91.0
1st capture with Panel B original probes	54.2	54.9

Panel C

samples	on-target (%)	on-target (%) with 300bp flanking region
1st capture with 50ng TBD probes	39.3	42.0
2nd capture with 50ng TBD probes	87.5	93.7
1st capture with Panel C original probes	73.3	79.8
